# Physical activity, organized sport participation and active transportation to school among Canadian youth by gender identity and sexual attraction

**DOI:** 10.24095/hpcdp.44.2.02

**Published:** 2024-02

**Authors:** Chinchin Wang, Gregory Butler, McKenna R.J. Szczepanowski, Marisol T. Betancourt, Karen C. Roberts

**Affiliations:** 1 Centre for Surveillance and Applied Research, Public Health Agency of Canada, Ottawa, Ontario, Canada; 2 Department of Epidemiology, Biostatistics and Occupational Health, McGill University, Montral, Quebec, Canada; 3 Centre for Clinical Epidemiology, Lady Davis Institute for Medical Research, Montral, Quebec, Canada; 4 School of Public Health Sciences, University of Waterloo, Waterloo, Ontario, Canada

**Keywords:** gender identity, sexual orientation, sexual and gender minorities, youth sports, exercise, physical activity, active transportation

## Abstract

**Introduction:**

Regular physical activity is associated with a wide range of health benefits in youth. While previous studies have identified disparities in physical activity among youth by gender identity and sexual attraction, these have seldom been explored in Canadian youth.

**Methods:**

Data from the 2019 Canadian Health Survey on Children and Youth were used to assess prevalence of and time spent in organized sports participation, total physical activity and active transportation by gender identity (non-cisgender vs. cisgender) among youth aged 12 to 17, and by sexual attraction (nonheterosexual attraction vs. heterosexual attraction) among youth aged 15 to 17.

**Results:**

There was no difference in average minutes of total physical activity per week between non-cisgender and cisgender Canadian youth. Non-cisgender youth (which represent 0.5% of the population) averaged significantly fewer minutes of organized sports per week than their cisgender counterparts. There was some evidence of increased active transportation to school among non-cisgender youth, but insufficient power to detect significant differences. Canadian youth reporting any nonheterosexual attraction (which represent 21.2% of the population, including mostly heterosexual youth) were less likely to be regularly physically active and participate in organized sports than youth reporting exclusive heterosexual attraction. Differences were larger among males than females. Males reporting nonheterosexual attraction were more likely to use active transportation to get to school than their heterosexual counterparts.

**Conclusion:**

Non-cisgender youth and youth reporting nonheterosexual attraction tended to participate less in organized sports than their counterparts, but may have engaged in more active transportation. Mitigating the barriers associated with sport participation could increase physical activity among these groups.

**Highlights**

- Non-cisgender youth in Canada had lower levels of participation in organized sports than cisgender youth; however, they engaged in similar levels of total physical activity.
- Nonheterosexual-attracted youth in Canada had lower levels of participation in organized sports and total physical activity than heterosexual-attracted youth.
- Efforts are needed to minimize barriers associated with organized sport participation for non-cisgender and nonheterosexual-attracted youth.

## Introduction

Regular physical activity is associated with a wide range of health benefits, including prevention of chronic diseases and improved well-being.^1^ The *Canadian 24-Hour Movement Guidelines for Children and Youth (ages 5–17 years)* recommend that children and youth obtain an average of at least 60 minutes of moderate-to-vigorous physical activity (MVPA) per day involving a variety of aerobic activities for optimal health benefits.^2^ The most recent device-measured national data from the 2018 to 2019 Canadian Health Measures Survey show that 35.6% of youth aged 12 to 17 years meet this recommendation.^3^ Lower intensities of physical activity (i.e. light physical activity; LPA) may also be beneficial for health. Research has shown strong, consistent associations between total physical activity (MVPA and LPA) and improved cardiovascular health, physical fitness and bone health, as well as lower adiposity.^4^

Identifying subpopulations that are less physically active can inform strategies and policies for health promotion. Gender identity and sexual attraction are important determinants of health. Various health and social inequities have been found for non-cisgender (i.e. identifying as a gender different from their sex at birth) and nonheterosexual (i.e. not exclusively attracted to the opposite gender) individuals compared to their cisgender (i.e. identifying as the same gender as their sex at birth) and heterosexual (i.e. exclusively attracted to the opposite gender) counterparts, respectively.^5^ Studies conducted in the US, UK, New Zealand and Spain have identified lower levels of physical activity and participation in organized sports among non-cisgender versus cisgender youth,^6^-^9^ as well as among nonheterosexual versus heterosexual youth.^9^-^17^ The magnitude of these differences varies by sex, with larger differences in physical activity and sports participation among cisgender and non-cisgender males than between cisgender and non-cisgender females.^10^

To the best of our knowledge, the only representative study assessing physical activity and sports participation by gender identity or sexual attraction among Canadian youth was based on the 1998 to 2013 British Columbia Adolescent Health Surveys. It found that males identifying as “mostly” heterosexual, bisexual or “mostly” or “completely” homosexual were consistently less likely to participate in both organized and nonorganized sports and physical activity compared to “completely” heterosexual males.^18^ Meanwhile, bisexual and “mostly” or “completely” homosexual females were less likely to participate in nonorganized sports and physical activity than heterosexual females, and bisexual females were less likely to participate in organized sports than heterosexual females.^18^

The objective of our study was to present current evidence by using nationally representative data from the 2019 Canadian Health Survey on Children and Youth to assess total physical activity, organized sports participation and active transportation use among Canadian youth (i.e. adolescents aged 12 to 17 years) by gender identity and sexual attraction.

## Methods

***Data source***

The data source for this study was the 2019 Canadian Health Survey on Children and Youth (CHSCY), a cross-sectional survey conducted by Statistics Canada. Data collection occurred between 11 February and 2 August 2019. The CHSCY surveyed a nationally representative sample of children and youth aged 1 to 17 years. Those living on First Nation reserves and other Indigenous settlements, those living in foster homes and the institutionalized population were excluded. The sampling frame consisted of beneficiaries of the Canadian Child Benefit, which covers 98% of the Canadian population aged 1 to 17 years in all provinces and 96% in all territories.

This study was limited to youth aged 12 to 17 years, which is a restricted age range in comparison to other (e.g. World Health Organization) definitions of youth. The survey was administered using electronic questionnaires or telephone interviews. All youth were asked about their gender and physical activity behaviours. Only youth aged 15 to 17 were asked about sexual attraction.

There were 11077 respondents aged 12 to 17 in the 2019 CHSCY (5301 aged 15 to 17). The response rate was 41.3%. Survey weights were provided by Statistics Canada to account for sampling and nonresponse and generate nationally representative estimates. Briefly, each respondent is assigned a weight based on design and adjustment factors that corresponds to the number of persons in the entire population that are represented by that respondent. Further details regarding weighting are provided online by Statistics Canada.^19^,^20^ Each analysis was restricted to those with complete data for physical activity behaviours, leaving 11064 respondents (99.9% of respondents aged 12 to 17) for gender identity, and 5254 respondents aged 15 to 17 (98.1% of respondents aged 15 to 17) for sexual attraction.

***Measures***

The exact wording for each survey question is provided online by Statistics Canada.^21^ The measures related to sex, gender and sexual attraction used in this study were based on available data and current statistical standards.^22^ Definitions and available measures are continually evolving and may not necessarily align with previous or future research. 

**Sex**

Youth aged 12 to 17 were asked, “What was your sex at birth? Sex refers to sex assigned at birth.” The response options were “male” and “female.”

**Gender identity**

Youth aged 12 to 17 were asked, “Gender refers to current gender which may be different from sex assigned at birth and may be different from what is indicated on legal documents. What is your gender?” The response options were “male,” “female” and “or please specify.” Youth who identified as a gender other than male or female were classified as “nonbinary.”

**Cisgender/non-cisgender**

Youth who identified as the same gender as their sex at birth were classified as “cisgender.” Youth who identified as a gender other than their sex at birth, including those considered “nonbinary,” were classified as “non-cisgender.”

**Sexual attraction**

Youth aged 15 to 17 were asked whether they were “only attracted to males”; “mostly attracted to males”; “equally attracted to females and males”; “mostly attracted to females”; “only attracted to females”; or “not sure.” Youth were classified as having “heterosexual attraction” if they identified as male gender and were attracted only to females or identified as female gender and were only attracted to males; or as having “nonheterosexual attraction” if they were attracted to the same gender, attracted to both males and females, or not sure, or if they identified as nonbinary gender. These classifications have been used in other studies.^23^,^24^

**Physical activity**

Individuals were asked for the total amount of time they spent participating in physical activity in which they sweated at least a little or breathed harder, as well as the amount of time they actually spent sweating or breathing harder for each of the past 7 days. Response options were in 15-minute increments (no time, 15 minutes or less, 30 minutes, 45 minutes, 1hour, etc., up to 7 hours or more). Those who answered 15 minutes or less or 7 hours or more were assigned a time of 15 minutes or 7 hours, respectively, for that day. The total time across all 7 days was used to calculate the average minutes of physical activity per day, since sensitivity analyses showed that these estimates were more in line with device-measured MVPA for Canadian youth than the time spent sweating or breathing harder, although they may include both MVPA and LPA.^3^ Individuals were categorized as averaging ≥60 minutes of physical activity per day versus averaging <60 minutes per day, which is the threshold used in the *Canadian 24-Hour Movement Guidelines for Children and Youth (ages 5–17 years)*.^2^

**Sports participation**

Individuals were asked whether they participated in sport or physical activity with a coach or instructor in the past year and past 7 days. Individuals were also asked for the total amount of time they spent participating in sport or physical activity in the past 7 days, which was divided by 7 to obtain an average sport participation time per day.

**Active transportation**

Individuals were asked whether they walked, biked or used another active way to get to school in the past 7 days, and the amount of time they spent using each of these modes of transportation. The amount of time spent using each mode was summed and divided by 7 to obtain an average active transportation time per day.

***Statistical analyses***

Descriptive statistics were used to calculate percentages, means and 95% confidence intervals (95% CIs) for gender identity (cisgender/non-cisgender) and sexual attraction overall, stratified by gender, and for physical activity, sports participation and active transportation indicators, stratified by cisgender/non-cisgender and heterosexual attraction/nonheterosexual attraction. Distributions were also calculated excluding those reporting “not sure” attraction as a sensitivity analysis. All percentages and means were calculated using survey weights to be nationally representative; 95% CIs were calculated using bootstrap weights. Two-tailed Wald chi-square tests were used to assess differences in means and percentages between groups under a statistical significance level of 0.05. Analyses were conducted in SAS Enterprise Guide version 7.1 (SAS Institute Inc., Cary, NC, US).

## Results

***Gender identity and physical activity***

Based on self-reported sex at birth and gender, 0.3% of youth aged 12 to 17 were classified as nonbinary and 0.5% as non-cisgender ( Table 1). All estimates for nonbinary and non-cisgender youth should be interpreted with caution due to their small sample size.

**Table 1:** Gender identity of study participants, youth aged 12 to 17 years, 2019 Canadian Health Survey on Children and Youth (N = 8418)

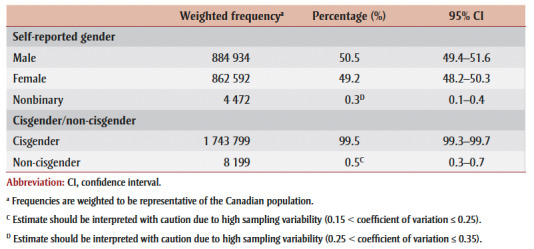

Physical activity measures by gender identity are presented in Table 2. Average minutes of physical activity per week did not differ between non-cisgender and cisgender youth. The percentages of non-cisgender youth who averaged 60 minutes or more of physical activity per day and who participated in organized sports in the past week were unreportable due to high sampling variability. Among non-cisgender youth, 48.5% participated in organized sports in the past year, compared to 67.3% of cisgender youth. Non-cisgender youth also averaged significantly fewer minutes of organized sport participation per week (96 minutes vs. 214 minutes). Finally, 48.0% of non-cisgender youth used active transportation to get to school, averaging 335 minutes per week, compared to 29.2% of cisgender youth, averaging 164 minutes per week. These differences were not significant.

**Table 2:** Physical activity measures for cisgender and non-cisgender youth aged 12 to 17 years, 2019 Canadian Health Survey on Children and Youth (N = 8418)

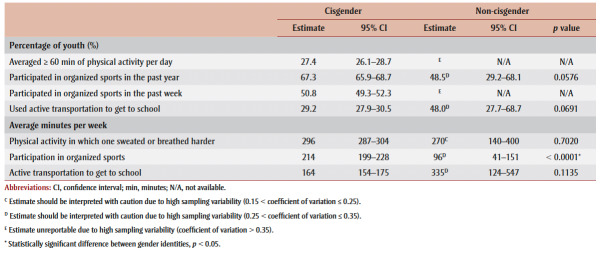

***Sexual attraction and physical activity***

Among youth aged 15 to 17, 78.8% reported heterosexual attraction while 21.2% reported nonheterosexual attraction (17.4% attracted to the same or both genders and 3.8% not sure of their attraction; Table 3). Females were more likely to report nonheterosexual attraction than males.

**Table 3:** Sexual attraction of study participants, youth aged 15 to 17, 2019 Canadian Health Survey on Children and Youth (N = 3963)

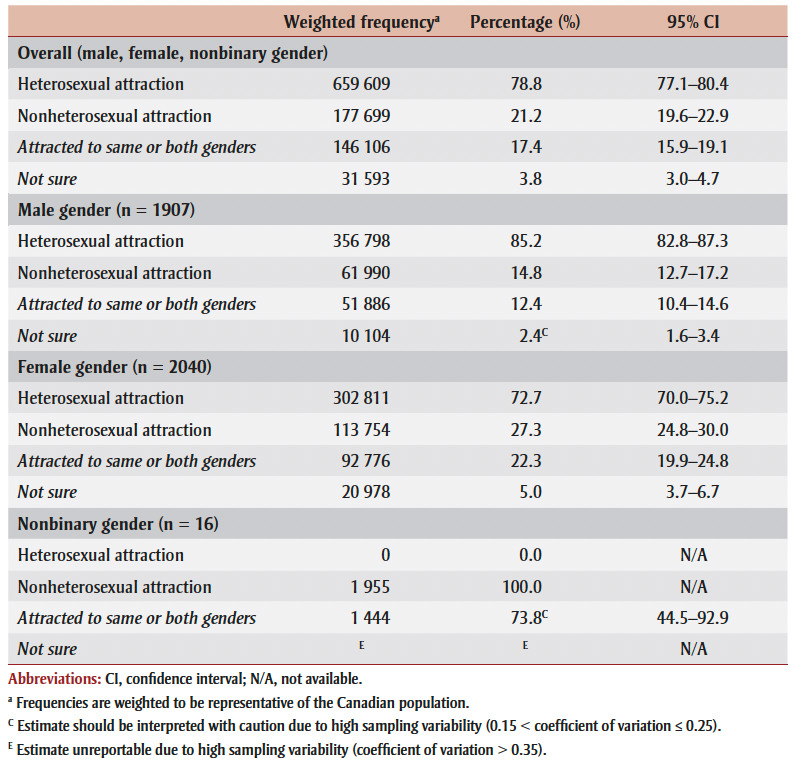

Physical activity measures by heterosexual/nonheterosexual attraction are presented in Table 4. Overall, youth reporting nonheterosexual attraction were less likely to average 60 minutes or more of physical activity per day (16.8% vs. 27.3%) and less likely to participate in organized sports in the past week (33.1% vs. 45.1%) and year (49.2% vs. 62.8%) than youth reporting heterosexual attraction. Youth reporting nonheterosexual attraction spent fewer weekly minutes being physically active (225 minutes vs. 284 minutes) and participating in organized sports (130 minutes vs. 216 minutes) than youth reporting heterosexual attraction; however, they spent more weekly minutes using active transportation to get to school (169 minutes vs. 126 minutes) than their heterosexual counterparts. While there was no difference in the overall percentage of youth using active transportation to get to school by sexual attraction, males reporting nonheterosexual attraction were more likely to use active transportation than those reporting heterosexual attraction. Males reporting nonheterosexual attraction also spent more time in active transportation to get to school per week, while achieving fewer minutes of organized sport participation. These differences were not apparent among females. There were no differences in trends when youth reporting “not sure” were excluded as a sensitivity analysis (data not shown).

**Table 4:** Physical activity measures by sexual attraction, youth aged 15 to 17 years, 2019 Canadian Health Survey on Children and Youth (N = 3963)

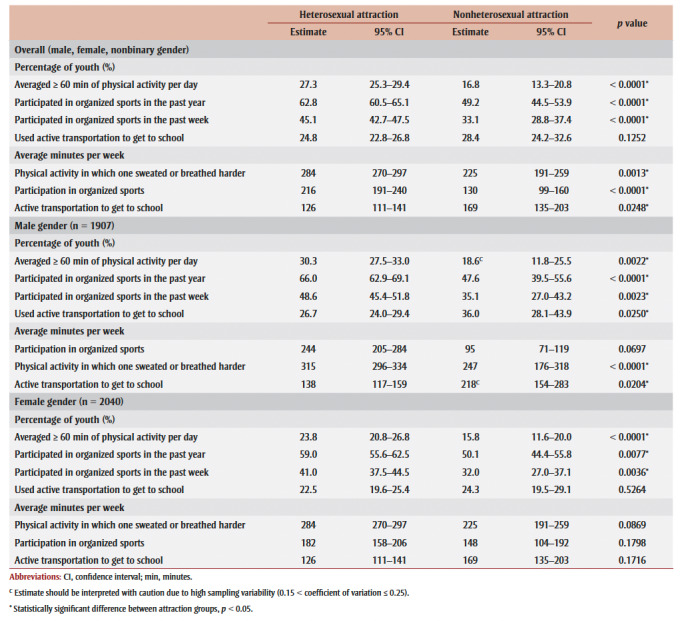

## Discussion

***Gender identity and physical activity***

To our knowledge, this was the first study to explore differences in measures of physical activity by gender identity among Canadian youth aged 12 to 17 years. Non-cisgender individuals made up a small percentage (0.5%) of the study population. There was no difference in the average weekly minutes of physical activity between non-cisgender and cisgender youth. The percentage of youth averaging 60 minutes or more per day of physical activity could not be compared in the two groups due to high sampling variability. This finding contrasts with existing research that found non-cisgender US high school students were less likely to be physically active for 60 minutes or more per day than cisgender students.^6^

Non-cisgender youth achieved significantly fewer minutes of organized sports participation per week than cisgender youth. Further, only half of non-cisgender youth participated in organized sports in the past year, compared to two-thirds of cisgender youth. This finding is in line with existing research from the United States^6^,^7^ and Spain;^8^ however, there was no difference in the likelihood to play sports between non-cisgender and cisgender youth in a 2014 survey of US high school students.^16^

Disparities in organized sport participation may be attributed to structural discrimination, such as policies that exclude transgender athletes from competitive sports.^25^-^27^ They may also be attributed to non-cisgender youth feeling uncomfortable or unsafe in organized sport environments, particularly in gender-segregated spaces (e.g. locker rooms).^10^,^16^,^27^-^29^ Non-cisgender youth may find ways to be physically active outside of organized sports. For instance, some non-cisgender respondents in a study of American youth noted that they preferred individual sports and physical activities (e.g. biking, rock climbing) to team sports.^27^ Nonetheless, participation in organized sports during adolescence contributes to health, not only through the benefits associated with regular physical activity, but through improved well-being and social development.^30^ Further efforts should be made to understand and mitigate the barriers associated with participation in organized sports.

Nearly half of non-cisgender youth used active transportation to get to school, compared to one-third of cisgender youth. Further, the average weekly minutes in active transportation to get to school among non-cisgender youth was over double that of cisgender youth. However, findings were not significant due to the small sample size.

Previous studies on active transportation use by gender identity were limited to adults. A US study found that non-cisgender college students engaged in active transportation more frequently than their cisgender counterparts, although findings were similarly limited by sample size.^31^ A study conducted in US adults suggested discomfort among non-cisgender individuals using public transit due to discrimination or abuse.^32^ Non-cisgender youth may similarly experience discomfort using public transit or school buses, and engage in active transportation to school as an alternative.^33^ Alternatively, non-cisgender youth may prefer active transportation as a way to be physically active over organized sports.^27^ Regardless of the reasons behind the observed differences between non-cisgender and cisgender youth, active transportation should be promoted for all youth as a way to be physically active and obtain the related health benefits.^34^

Overall, these results show different modes of attaining physical activity for non-cisgender and cisgender youth aged 12 to 17. However, the small number of non-cisgender youth in the study sample impacted our ability to identify differences between these groups where they might exist, especially where absolute percentages suggest differences between groups.

***Sexual attraction and physical activity***

One-fifth of youth aged 15 to 17 reported nonheterosexual attraction (attracted to the same or both genders, or not sure of their attraction). Youth reporting nonheterosexual attraction were less likely to average 60 minutes or more of physical activity per day, regardless of gender. They also averaged 60 fewer minutes of physical activity per week than their heterosexual counterparts. These findings are in line with existing Canadian research demonstrating lower levels of physical activity (organized or non-organized) among nonheterosexual youth in British Columbia,^18^ as well as research from the UK^13^ and US.^11^,^12^,^14^,^15^,^17^

Youth reporting nonheterosexual attraction were less likely to participate in organized sports in the past week and year than youth reporting heterosexual attraction. These differences were larger among males than females. This finding is consistent with those of the BC Adolescent Health Surveys, which have consistently shown lower participation in sports and physical activity with a coach among nonheterosexual youth compared to heterosexual youth, with larger differences among males.^18^ Studies from the US have found lower levels of participation in team sports and school sports among nonheterosexual youth, particularly among males.^14^,^16^,^17^ Many nonheterosexual youth avoid organized sports due to bullying and discrimination from peers and staff, and because they feel unsafe or uncomfortable in sport settings (e.g. physical education class, locker rooms).^10^,^16^,^27^,^29^,^35^

The differences between males and females may be partially attributed to the perception of nonheterosexual females as more masculine and therefore more competent in sport than their heterosexual counterparts.^10^ In addition, nonheterosexual male youth tend to experience greater bullying victimization than their female counterparts across sport and other environments.^25^,^36^,^37^

However, differences in total physical activity were smaller than differences in organized sport participation between nonheterosexual- and heterosexual-attracted males, suggesting that nonheterosexual-attracted males make up for lower levels of organized sport participation with other physical activity (e.g. nonorganized sports, active transportation). For instance, a study in the US found that lesbian, gay, bisexual, transgender and queer youth generally prefer individual sports (which are often nonorganized) to team sports.^27^

Males reporting nonheterosexual attraction were more likely to use active transportation to get to school and spent more time doing so than males reporting heterosexual attraction. There was no difference in active transportation use among females. Active transportation to school may be an important coping mechanism to avoid bullying behaviour on public transit or school buses.^38^ Studies have shown that nonheterosexual male youth are more likely to be bullied than their female counterparts,^36^,^37^ which may explain the gender difference in the uptake of active transportation. Otherwise, a US study found that youth with nonheterosexual attraction were less likely to have a driver’s license upon reaching young adulthood, which may be due to lower parental support in obtaining a license.^39^ Given that practising driving on routine trips to school is common among youth,^40^ the higher rates of active transportation to school among nonheterosexual males may also be attributed to lower likelihood of parental support for driving. Finally, males with nonheterosexual attraction may simply prefer active transportation as a way to be physically active over organized sports.^27^

Overall, these results point to lower levels of physical activity and organized sport participation among nonheterosexual-attracted youth compared to heterosexual-attracted youth aged 15 to 17. These discrepancies were larger among males than females. Our results were in line with Canadian and international studies demonstrating decreased physical activity and sport participation among nonheterosexual youth. Efforts should be made to decrease the barriers associated with physical activity and sport participation. This might be done by implementing antibullying programs in sport, promoting nonheterosexual athletes as role models, encouraging peer and familial support and introducing youth to a wider range of physical activities in school.^14^,^41^

***Strengths and limitations***

This study assessed multiple measures of physical activity (total physical activity, organized sport participation and active transportation to school) by gender identity and sexual attraction among Canadian youth. It was the first study, to our knowledge, to examine differences in each physical activity measure by gender identity among Canadian youth, and in active transportation to school by sexual attraction. Future studies would benefit from examining additional measures of physical activity, such as participation in nonorganized sports, participation in different sport settings (i.e. group vs. individual, types of sports) and the meeting of muscle- and bone-strengthening recommendations in the *Canadian 24-Hour Movement Guidelines*, among these subpopulations.^2^

This study had several limitations. Despite the use of a large sample of Canadian youth, the low prevalence of non-cisgender individuals meant there was insufficient power to detect significant differences for most measures and to examine behaviours of nonbinary and transgender youth separately. Several estimates could not be reported due to high sampling variability. There was insufficient sample size to report estimates for detailed sexual attraction categories (e.g. males attracted only to males; nonbinary attracted only to males). The sample size was also insufficient to explore how other socioeconomic characteristics (e.g. ethnocultural background, household income) intersect with gender identity and sexual attraction in the assessment of physical activity. These findings are important to note for future surveys; researchers may wish to increase sample sizes or oversample non-cisgender and nonheterosexual-attracted youth in order to report more detailed breakdowns.

Additionally, the wording of certain questions limited our reporting. Data on gender were collected using biological terms (“female” and “male”), and all youth who reported a gender other than female or male were classified as nonbinary. It was not possible to determine breakdowns for specific gender identities, such as Two-Spirit or queer. Further, the questionnaire did not specify whether sexual attraction towards males and/or females was based on gender or sex. In this study, we assumed that attraction was based on gender rather than sex, which may not be the case for all respondents.

## Conclusion

Gender identity and sexual attraction are important predictors of physical activity among Canadian youth. Our findings demonstrate different modes of engaging in physical activity between non-cisgender and cisgender youth aged 12 to 17 years, with similar total physical activity yet lower organized sport participation and some evidence of higher active transportation use among non-cisgender youth. We also found lower levels of organized sport participation and total physical activity among Canadian youth aged 15 to 17years reporting nonheterosexual attraction compared to those reporting heterosexual attraction, particularly among males, but increased active transportation only among males. Mitigating the barriers associated with participation in organized sports while additionally promoting active transportation could increase physical activity among all youth.

## Conflicts of interest

The authors have no conflicts of interest to disclose.

## Authors’ contributions and statement

CW, GB, MTB, KCR—conceptualization.

CW—methodology, formal analysis, writing—original draft.

CW, GB, MRJS, MTB, KCR—writing—review & editing.

The content and views expressed in this article are those of the authors and do not necessarily reflect those of the Government of Canada.
